# Supplementary material for: PET-MAD as a lightweight universal interatomic potential for advanced materials modeling
Source: Nat Commun. 2025 Nov 27;16:10653. doi: 10.1038/s41467-025-65662-7 (PMC12660877; doi:10.1038/s41467-025-65662-7)
Supplement: Supplementary file 1 — Supplementary Information [file 41467_2025_65662_MOESM1_ESM.pdf]

PET-MAD as a lightweight universal interatomic potential for advanced materials modeling. Supplementary Information.

Arslan Mazitov[1*†], Filippo Bigi[1†], Matthias Kellner[1], Paolo Pegolo[1], Davide Tisi[1], Guillaume Fraux[1], Sergey Pozdnyakov[1], Philip Loche[1], Michele Ceriotti[1*]

[1]Laboratory of Computational Science and Modeling, Institut des Matériaux, École Polytechnique Fédérale de Lausanne, 1015, Lausanne, Switzerland.

*Corresponding author(s). E-mail(s): arslan.mazitov@epfl.ch; michele.ceriotti@epfl.ch;
†These authors contributed equally to this work.

1 MAD dataset details

Name	Description	# Structures
MC3D	Bulk crystals from the Materials Cloud 3D crystals database [1]	33,596
MC3D-rattled	Rattled analogs of the original MC3D crystals, with Gaussian noise added to all atomic positions	30,044
MC3D-random	Artificial structures obtained by replacing the atomic species of a few MC3D structures with a random sampling from the list of all 85 elements	2,800
MC3D-surface	Surface slabs generated from the MC3D structures by cleaving the crystal along a random crystallographic plane with low Miller index	5,589
MC3D-cluster	Nanoclusters, generated by cutting a random atomic environment of 2 to 8 atoms from a random crystal sampled from the MC3D and MC3D-rattled subsets	9,071
MC2D	Two-dimensional crystals from the Materials Cloud 2D crystals database [2]	2,676
SHIFTML-molcrys	A curated subset of the SHIFTML molecular crystals structures [3], that are in turn sampled from the Cambridge Structural Database [4] including both relaxed and thermally-distorted configurations	8,578
SHIFTML-molfrags	Neutral molecular fragments extracted from the SHIFTML dataset [5]	3,241

Supplementary Table 1 Overview of the MAD dataset structure. The dataset contains a total of 95,595 structures of 85 elements (with atomic numbers ranging from 1 to 86, excluding Astatine) and consists of eight subsets of various chemical and structural natures.

2 Hyperparameters optimization

To obtain the optimal model in terms of accuracy and computational speed, we performed a grid search over the most important hyperparameters that define the architecture of the model. The hyperparameters and the values they were ranged in are provided below:

1. The cutoff radius of the model (R_{cut}) - [4.0, 4.5, 5.0, 5.5]
2. Number of message-passing (MP) layers (N_{GNN}) - [1, 2, 3, 4]
3. Number of transformer layers in each MP layer (N_{trans}) - [1, 2, 3, 4]
4. Dimensionality of the hidden space (d_{PET}) - [64, 128, 256]
5. Number of heads in the multi-head attention layers (N_{heads}) - [4, 8]

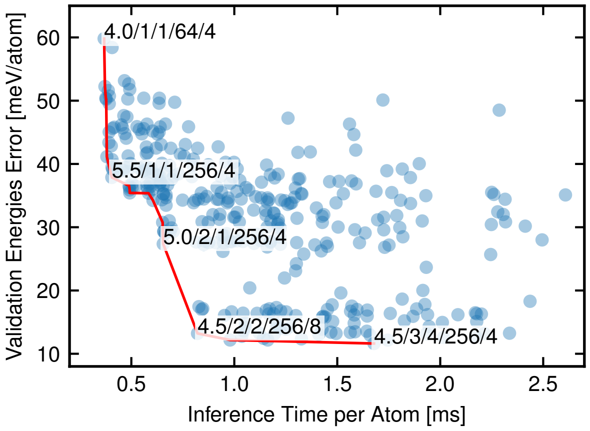

Supplementary Figure 1 Pareto frontier of the PET-MAD models performance with different architectures. Each architecture is represented as a single point on a scatterplot. Inference time per atom on a single NVIDIA GH200 GPU with a batch size of 1 is plotted along the x-axis. The mean absolute error (MAE) in predicting energies on the validation set is plotted along the y-axis. The Pareto frontier is drawn as a solid red line. A few selected architectures are highlighted using the following notation: $R_{\text{cut}}/N_{\text{GNN}}/N_{\text{trans}}/d_{\text{PET}}/N_{\text{heads}}$. The optimal model has hyperparameters of 4.5/2/2/256/8.

For each combination of the hyperparameters a separate training was performed, and model accuracy and inference time on a validation set was evaluated thereafter using a single NVIDIA GH200 GPU with a batch size of 1 – which is representative of the performance of the model for molecular dynamics. The resulting data was plotted on a single scatter plot (Suppl. Figure 1), and the Pareto-frontier construction was used to find the optimal set of hyperparameters. Based on this analysis, we conclude that the optimal PET-MAD model has a cutoff radius of 4.5 Å, 2 MP layers with 2 transformer layers each, 256 neurons in the hidden space and 8 heads in the multi-head attention layers.

3 Details of benchmarking subsets selection

We compared the accuracy of PET-MAD against four recent universal machine learning interatomic potentials - MACE-MP-0 L, MatterSim-5M, Orb-v2, and SevenNet-l3i5 - on popular atomistic ML datasets: MPtrj, Matbench Discovery, Alexandria, SPICE, MD22, and OC2020 S2EF. For each dataset (including MAD), we prepared a small subset of structures that were recalculated using DFT settings consistent with each model's training data. A description of the dataset is provided in Supplementary Table 2.

The MAD benchmark consists of 360 structures selected by sampling 50 random structures from each MAD test subset, recalculated with MPtrj DFT settings, and cleaned of non-converged structures and outliers. The MPtrj benchmark is based on the MACE-MP-0 validation subset, reduced to 136 structures after removing four 1D

Name	Description
MAD	The dataset developed in this work
MPtrj	Relaxation trajectories of bulk inorganic crystals dataset from Ref. ?
Matbench	The dataset of single-element substitutions on bulk inorganic crystals from Ref. 8
Alexandria	Relaxation trajectories of bulk inorganic crystals as well as 2D, 1D systems from Ref. ?
OC2020 (S2EF)	Molecular relaxation trajectories on catalytically active surfaces from Ref. 6
SPICE	Drug-like molecules and peptides from Ref. ?
MD22	Molecular dynamics trajectories of peptides, DNA molecules, carbohydrates and fatty acids from Ref. 7

Supplementary Table 2 Overview of the benchmarks used for evaluating PET-MAD against other universal MLIPs.

wire structures. The Matbench Discovery benchmark contains 555 structures, randomly sampled from the original WBM dataset [8] - a part of the Matbench Discovery, which addresses the structural stability of inorganic crystals with random elemental substitutions. We didn't include the structures with lanthanides and actinides to this subset in order to balance the effect of low coverage of these elements in the MAD dataset compared to other datasets like MPtrj. The OC2020-S2EF benchmark consists of 78 structures, where 100 structures were first sampled from the OC2020-S2EF training dataset and then cleaned of non-converged cases. The SPICE benchmark contains 99 structures, where 100 structures were randomly sampled neutral molecules from the SPICE dataset, and later cleaned of non-converges ones. The Alexandria benchmark includes 200 structures: 50 were randomly sampled from Alexandria-2D and Alexandria-3D-gopt, and 100 from Alexandria-3D. The MD22 benchmark consists of 134 structures, where 25 structures were first randomly sampled from each of the seven subsets of the original MD22 dataset (Ac-Ala3-NHMe, AT-AT, DHA, Stachyose, AT-AT-CG-CG, Buckyball-Catcher, double-walled-nanotube), and then cleaned of non-converged cases.

4 Assessing PET-MAD on Matbench Discovery benchmark

As discussed throughout this work, it is crucial to maintain consistency in the level of DFT theory in the training set of the model. Lack of consistency in training data can introduce systematic errors to the model predictions, which can sometimes go up to 30 meV/atom (see Suppl. Figure 3 for details). It is equally important to keep the

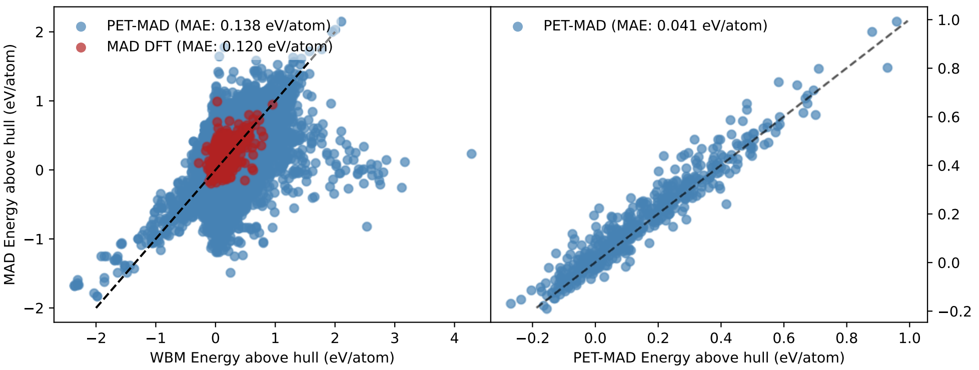

Supplementary Figure 2 Results of the PET-MAD benchmarking on the WBM dataset. Right panel shows the comparison of the energies above hull of a subset of structures from the WBM dataset [8], recomputed with DFT using MAD settings (MAD DFT, red dots), and predicted with PET-MAD (blue dots). The mean absolute error (MAE) in predicting the WBM values of the energy above hull in eV/atom is presented in the legend. This comparison demonstrates the upper limit of accuracy of any MAD-trained on the Matbench-Discovery benchmark upon using non-consistent DFT settings, as the difference in baseline DFT energy between Matbench and MAD settings reaches 120 meV/atom. Left panel shows the comparison of the PET-MAD predictions of the energy above hull against the consistent reference, recomputed with MAD settings. The resulting error decreases from 138 to 41 meV/atom, revealing the actual accuracy of the model.

consistency while assessing the accuracy on the model on benchmarks, like the popular Matbench Discovery [9]. Suppl. Figure 2 demonstrates the results of the PET-MAD assessment on the WBM dataset [8] (a part of the Matbench Discovery) in predicting the energies above hull. When comparing the predictions against the default WBM values, PET-MAD yields a large error of around 140 meV/atom. However, this error is almost entirely explained by the significant discrepancy of 120 meV/atom in the underlying DFT reference, as demonstrated by comparing the WBM values to those recomputed using the MAD dataset settings on a subset of the WBM crystals. This value essentially sets an upper limit on the accuracy of any MAD-trained model when compared to data obtained with inconsistent (in this case - WBM) DFT settings. However, while using the consistent level of theory, one can significantly improve the models' accuracy: in the left panel of the Suppl. Fig. 2 we demonstrate the actual accuracy of the PET-MAD model if compared to the consistent DFT reference: the MAE drops from 138 meV/atom to 41 eV/atom.

This final MAE value was used to address another important property of the universal MLIPs - their data-efficiency. For all the reference in the main text, we calculated the MAE value in predicting the energy above hull based on the same WBM subset, while utilizing the default WBM energy values for all the models except PET-MAD.

Therefore, since there is no straightforward way to account for significant discrepancies in the baseline DFT data in the Matbench Discovery benchmark, we decided not to add PET-MAD to the models list on the benchmark website. The resulting

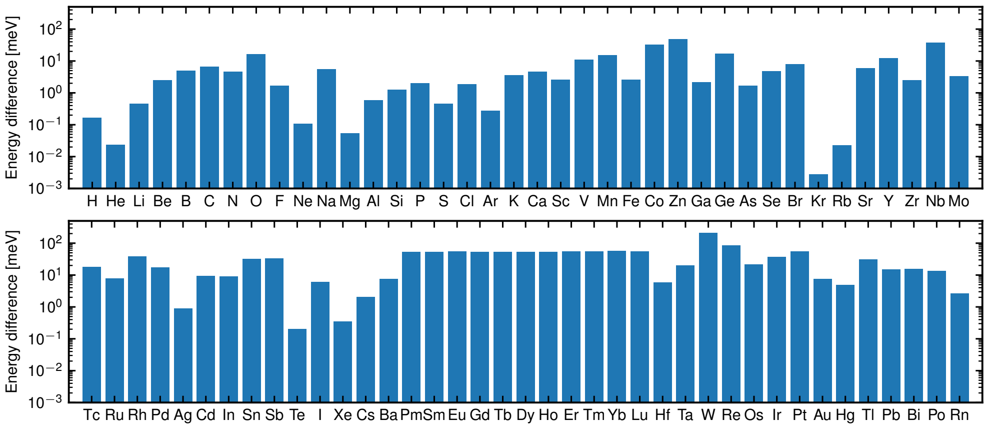

Supplementary Figure 3 Effect of the DFT settings on the energies of isolated atoms. Absolute difference in energies of isolated atoms calculated with MAD dataset settings and recommend SSSP values of the plane waves basis set and charge density cutoffs.

accuracy numbers would not be representative due to the lack of consistent DFT reference. We also didn't include the phonon properties related part of the Matbench Discovery benchmark in this discussion, as we instead performed a more systematic study of the phonon properties in Supplementary Section 9.

5 Effect of *ab initio* calculations settings convergence

One of the main features of the MAD dataset developed in this work is its high internal consistency in the DFT settings used. This implies the use of extremely high cutoff values for the wavefunction basis set (110 Ry) and charge density (1360 Ry), regardless of the considered system and set of elements. These values effectively are consistent with the most restrictive recommended settings that are provided in the SSSP pseudopotential library [10] (which range from 30 / 120 Ry to 90 / 1080 Ry for the wavefunctions basis set / charge density cutoffs, respectively) for the elements included in the MAD data set (except for Radon, which has the recommended cutoffs of 120 Ry and 960 Ry, respectively). To explain the necessity for this protocol (rather than that one would use in first-principles calculations, that usually involves choosing the most restrictive settings *within each structure*) we compare the energies of isolated atoms (which we use as a baseline in MAD dataset), calculated using MAD settings and the recommended SSSP settings for each element (Suppl. Figure 3). These results show that for certain elements the difference in energies between the recommended and MAD settings can reach 10-100 meV/atom, which is either comparable or higher than the typical error of PET-MAD. If the level of convergence depended on the composition of each structure, the energy contribution of each atom would not depend exclusively on the atoms within the receptive radius of the model, but on the global composition, which is unphysical and ultimately unlearnable. While it may be possible to carefully correct for these effects (that do not usually affect *relative energies* within

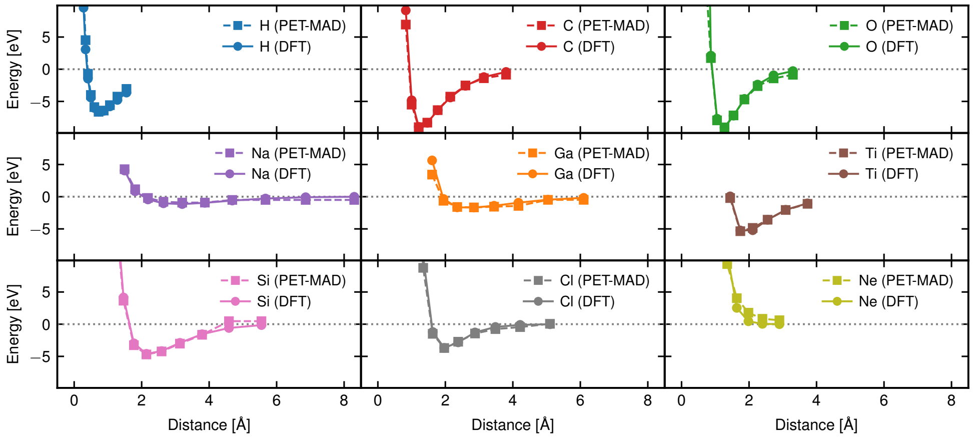

Supplementary Figure 4 Results of the PET-MAD model in predicting dimer curves. Energy dependence of elemental dimers on different interatomic distances for several elements, calculated with MAD DFT settings and the PET-MAD model. All dimers were treated as diatomic clusters and shared the DFT calculation protocol with the MC3D-cluster subset.

a fixed composition space) we prefer to avoid the inconsistency altogether, despite the substantial computational overhead in training-set construction.

6 Diatomic energy curves

Lack of training data in the region of small interatomic distances can lead to a phenomenon of *artificial dimers*, where a pair of atoms essentially merge into a single point in space due to no explicit repulsive part in the potential. One way to solve this problem is to include constraints in the model, which define the behavior at short interatomic distances. Another way is to make the model learn this behavior by adding the appropriate configurations with close atomic distances to the data set. In this work, we use a second approach while training the PET-MAD model, and it yields quantitatively accurate diatomic curves with correct repulsive behavior (Suppl. Figure 4). This is mainly due to the MAD dataset, which is diverse enough to provide the necessary data in the range of short interatomic distances. The set of distances on which we evaluated the energies of dimers is based on a logarithmic grid of ten points ranging from 0.9 to 5.0 of the corresponding element's covalent radius in angstroms.

7 Geometry optimization with PET-MAD

We evaluated PET-MAD for geometry optimization on the three materials from Sec. 9, namely, wurtzite BeO, zincblende BeTe, and rocksalt LiBr taken from the phononDB dataset. We used a simple optimization workflow using ASE calculators and the LBFGS optimizer with a default maximum force threshold of 0.05 eV/Å. To collect statistics, we performed 1000 optimization tasks per material, applying random displacements to atomic positions and lattice vectors from a normal distribution with

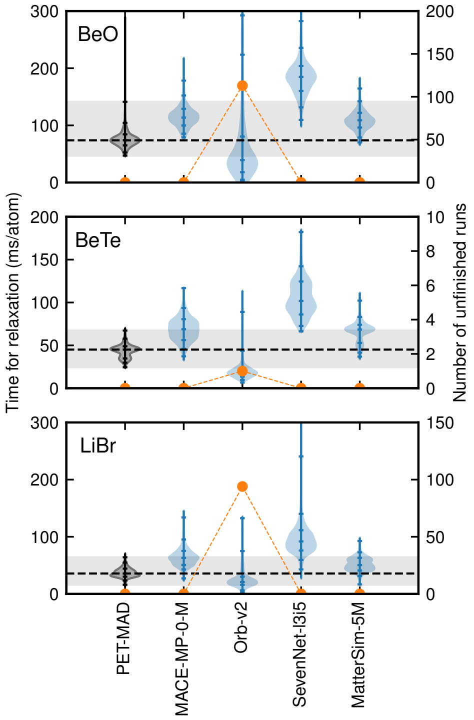

Supplementary Figure 5 Geometry optimization performance of a few selected universal MLIPs. Timing distributions for 1000 geometry optimization runs using the L-BFGS optimizer. The black dashed band represents PET-MAD timings within three σ from the median under the Gaussian approximation (0.135th to 99.865th percentile). The orange circles are the number of times the optimization runs did not complete.

zero mean and standard deviation of 0.1 Å. We measured the time to complete each optimization for the five universal MLIPs used in our analyses and show the time distributions in 5. We limited the maximum number of optimization steps to 100. PET-MAD timings are comparable to or faster than those of all conservative models tested. The non-conservative Orb-v2 is consistently faster but less stable, being the only model that failed to converge in up to 10% of the displacements.

8 Uncertainties of PET-MAD

Replacing expensive numerical computations with efficient statistical models is the centerpiece of this manuscript. Predictions of the universal force field are potentially uncertain with respect to the reference method it aims to approximate. Model uncertainties of universal force fields are dominated by epistemic contributions that arise from a lack of knowledge of the model about an area of the structural and compositional space that is only loosely covered by the training set. Given the vast compositional and structural space, any universal machine learning forcefield will suffer from areas of low coverage of training samples, especially since new, undiscovered

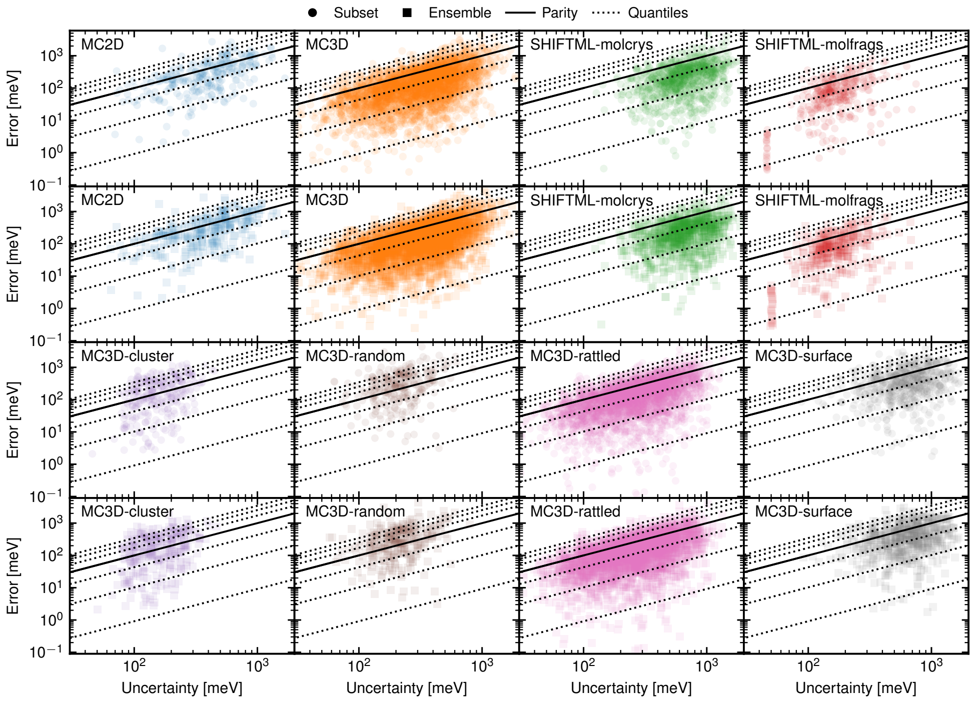

Supplementary Figure 6 Reliability of the uncertainty quantification of the PET-MAD model. Upper panels per color: Predicted vs. actual per structure error of the PET-MAD model on the MAD test set within the LLPR protocol. Lower panels per color: same error but after converting the LLPR uncertainty model to a shallow ensemble. A solid line shows parity in prediction ($y = x$), while dotted lines show the first, second, and third quantiles.

classes of material could never have been considered in the construction of the training set. Epistemic uncertainties are by no means a reason why universal forcefields are bound to fail from the outset; rather, it is necessary to manage them carefully, by equipping universal models with uncertainty quantification schemes, that indicate to the user when model predictions are uncertain and need to be treated with caution. Model errors also propagate to derived quantities, such as thermodynamic averages of observables.

Oftentimes model uncertainties in atomistic machine learning models are estimated using ensembles or committees of models, due to their conceptual simplicity and ease of implementation, requiring only to train a series of models and evaluating them. Albeit conceptually simple and easy to implement, full ensembling increases training and evaluation cost linearly with the number of models in the ensemble. The LLPR formalism [11] offers an alternative way to obtain cheap ensembles from a trained neural network. Within the LLPR method, predictive uncertainties are computed as

$$\sigma_i^2 = \alpha\, \mathbf{f}_i^\top (\mathbf{F}^\top \mathbf{F} + \varepsilon^2 \mathbf{I})^{-1} \mathbf{f}_i. \tag{1}$$

In this expression, σ_i is the uncertainty on the prediction relative to sample i, $\mathbf{f}_i$ are the latent features in the last layer of the neural network, $\mathbf{F}$ is a matrix whose rows correspond to each set of last-layer features for each structure in the training set, and ε is a small regularizer. Besides computing energy uncertainties at nearly no additional cost compared to the raw predictions, the LLPR also allows the sampling of a last-layer ensemble [11], whose uncertainties can be propagated numerically through arbitrarily difficult workflows, including molecular dynamics [12]. The GaAs calculations in the main text demonstrate how combining LLPR-sampled last-layer ensemble models with thermodynamic reweighting enables uncertainty quantification of PET-MAD predictions and propagate their uncertainties to thermodynamic averages at virtually no additional cost, at both training and inference time. The LLPR uncertainties, as well as those from an LLPR-derived last-layer ensemble of 128 members, are shown in Suppl. Figure 6 for energy predictions on the MAD test set. The predictions follow the expected distribution almost exactly (see, for example, Refs. [11] and [12] for a discussion of this type of plots), confirming the very high quality of PET-MAD uncertainties, at least within the training domain.

9 Uncertainty estimation for phonon dispersion curves

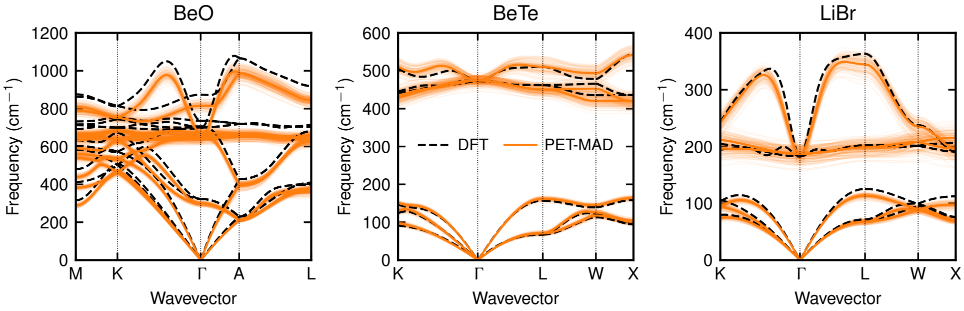

Supplementary Figure 7 Results of the PET-MAD benchmarking on the phononDB dataset. LLPR ensembles of phonon bands (orange, thin lines) for three representative structures of the phononDB dataset, compared with reference DFT results (black, dashed lines). Average values of the bands are reported denoted by orange, thick lines.

In addition to complex properties that require MD sampling, such as the melting temperature discussed in the main text, uncertainty can also be estimated for static properties like phonon band structures. In Suppl. Fig. 7, we present the LLPR ensemble of phonon bands for three representative materials from the PBEsol phononDB dataset [13]: wurtzite BeO, zincblende BeTe, and rocksalt LiBr, which were also analyzed in Ref. [14] using the MACE-MP-0 foundation model.

In the case of BeO, the optical bands appear slightly softer than in the reference calculations, which we checked to produce phonon bands consistent with calculations

Supplementary Table 3 Accuracies of the LoRA-finetuned models in predicting the energies (in meV/at.) and forces (in meV/Å) on the MAD test set. For the pre-trained PET-MAD errors are 15.1 meV/at. | 72.3 meV/Å.

LoRA Model	Energy MAE meV/at.	Forces MAE meV/Å
LPS	129.4	215.1
GaAs	78.8	134.5
HEA25S	91.1	228.9
Water	284.8	288.3
BTO	44.4	140.8
Succinic acid	144.4	191.1

done with MAD settings with a frequency root mean square deviation of 3 cm^{-1}. This behavior, however, is commonly observed for universal MLIPs, as shown in Ref. [15], and occurs for BeO as well as for the other materials presented here when using MACE-MP-0 [14]. The deviation is reflected in the UQ, which shows increased variance in the phonon band ensemble for optical modes.

10 Fine-tuning accuracies

For each simulation case presented in this work we trained a bespoke PET model from scratch, and compared it against the LoRA-finetuned version. While being equally accurate in predicting observables, the fine-tuned model retains a certain degree of accuracy on the base MAD dataset, which can be beneficial in certain computational setups. In Table 3, we list the mean absolute errors of each fine-tuned model in predicting the energies and forces on the base MAD test set. For reference, the general-purpose PET-MAD model yields errors of 15.1 meV/at. | 72.3 meV/Å on the MAD test set.

11 Learning curves

11.1 PET-MAD

Suppl. Figure 8 shows the learning curves of the PET-MAD model. Each training was performed on a fraction of MAD dataset, where a corresponding fraction of the structures was sampled from each subset, and then unified and shuffled. Even after training on only 20 % of the data, the model achieves a reasonable accuracy with a mean absolute error in predicting forces of the MAD test set of 134.7 meV/Å. With further increase in the training dataset size, the test MAE for both energies and forces gradually reduces. After reaching 60% of the training data, we observe that the errors on a train set start growing up, which means that the dataset is diverse enough that the model cannot overfit. This saturation is not an indication of intrinsic limitations of the PET architecture, but of the fact that - for the small size of the MAD dataset - the Pareto optimal architecture corresponds to a comparatively lightweight model that is best adapted to avoid overfitting. Lack of saturation in the test set indicates

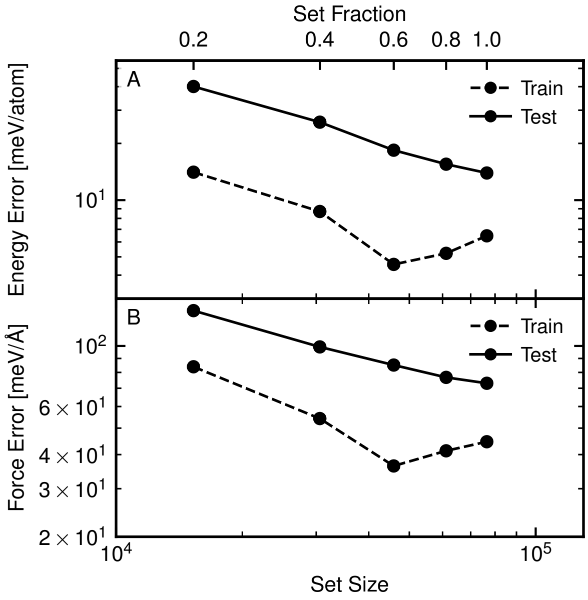

Supplementary Figure 8 Learning curves of the PET-MAD model. The random fraction of the full MAD training set used for training is plotted on a x-axis, and the mean absolute error in predicting energies and forces on a MAD test set is given on a y-axis.

that PET-MAD could still benefit from an increase in the train set size, and it would be easy, if needed, to obtain a more expressive model by increasing the number of GNN and transformer layers.

11.2 Ionic transport in lithium thiophosphate

Suppl. Figure 9 shows learning curves for the Li_3PS_4 dataset for three learning scenarios, i.e., training from scratch (orange), LoRA finetuning with a rank of 8 (blue), and complete fine-tuning of all the model parameters (green). The black dashed line corresponds to the MSE of PET-MAD on the LPS test set. The complete fine-tuned seems to outperform the others of about 20 %, possibly because of the reduced number of epochs required during fine-tuning to reach a small error with respect to a model trained from scratch. LoRA finetuning provides slightly higher errors on forces with respect to the bespoke PET model, however from the results in the main text seems that this small difference does not translate in a large difference in the values of the ionic conductivity.

11.3 Melting point of GaAs

Suppl. Figure 10 shows the learning curves for the Gallium Arsenide dataset, for the training of a bespoke PET model and LoRA and fully finetuned PET-MAD models, as well as the base accuracy of PET-MAD (black-dashed line).

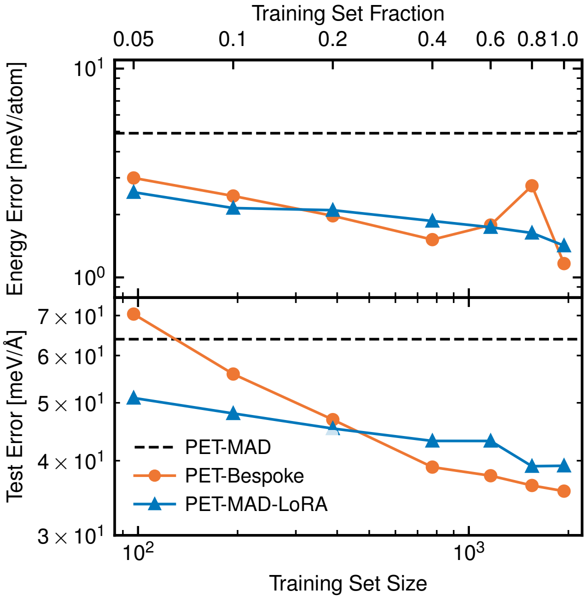

Supplementary Figure 9 Learning curves (forces) for the Li_3PS_4 dataset of Ref. [16]. The results of a bespoke model (orange line) and a LoRA-finetuned model (blue line) with rank 8 are compared against the general PET-MAD model (black dotted line).

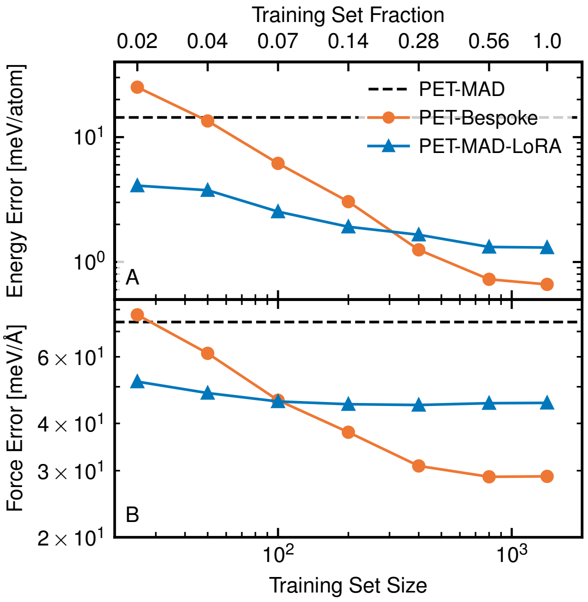

Supplementary Figure 10 Learning curves (forces) for the Gallium Arsenide dataset. The results of a bespoke model (orange line) and a LoRA-finetuned model (blue line) with rank 8 are compared against the general PET-MAD model (black dotted line).

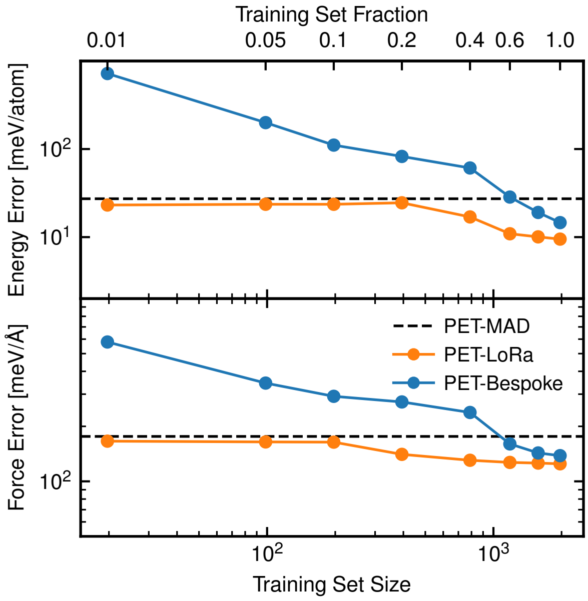

Supplementary Figure 11 Learning curves for the subset of the HEA25S dataset [17]. The results compare training a bespoke model from scratch (blue line) and LoRA finetuning (orange line) with LoRA rank 8 against the base PET-MAD model predictions error (black dotted line).

11.4 Surface segregation in high-entropy alloys

Suppl. Figure 11 shows the learning curves of three models, namely the bespoke PET model trained on a subset of the HEA25S dataset [17] from scratch, and two fine-tuned models, which start from the pre-trained PET-MAD weights: the fully fine-tuned and a LoRA-finetuned model. Both fine-tuned models show similar training behavior in a low-data regime with a fully funetuned one demonstrating better accuracy as the amount of training data increases. In contrast to other simulations cases done in this work, the bespoke model cannot achieve the same accuracy on a test set, compared to fine-tuned models. This is likely caused by undersampling in the HEA25S subset used for training, which only contains 1975 structures with 25 transition metals, while the original dataset has about 30,000 structures.

11.5 Quantum nuclear effects in liquid water

Suppl. Figure 12 shows learning curves for the liquid water dataset for three learning scenarios: (1) training from scratch, (2) LoRA finetuning with a rank of 8, (3) LoRA finetuning with a rank of 32. It can be seen that fine-tuning the PET-MAD model under these conditions is advantageous until approximately 20% of the entire training set (which consists of 1228 structures overall) is used. With more structures, the fine-tuned model is less accurate; this is partially remedied by increasing the rank of LoRA.

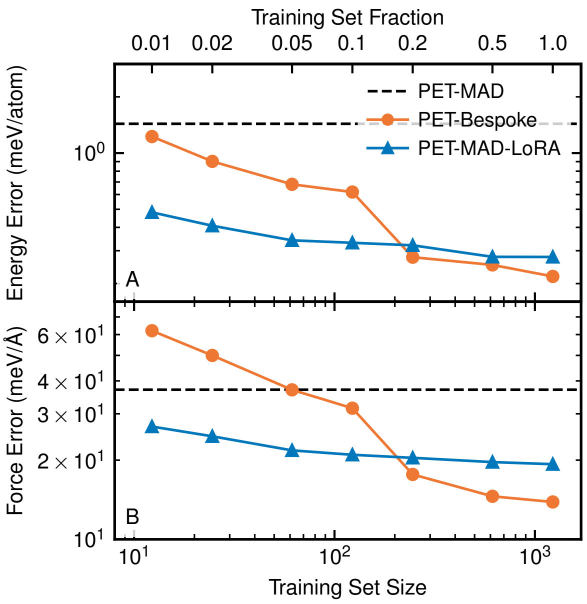

Supplementary Figure 12 Learning curves for the water dataset. The results compare training a bespoke model from scratch (blue line) and LoRA finetuning (orange line) with LoRA rank 8 against the base PET-MAD model predictions error (black dotted line).

11.6 Quantum nuclear effects in NMR crystallography

Suppl. Figure 13 shows learning curves for the succinic acid dataset, for all three learning scenarios.

For the computation of chemical shielding of succinic acid crystals, we construct an auxiliary model to predict chemical shieldings. We construct one linear model per central species from SOAP descriptors, computed with the featomic library. A parity plot of predicted and reference chemical shielding values is shown in Figure 14.

11.7 Dielectric response of barium titanate

Suppl. Figure 15 shows learning curves for the BTO dataset for three learning scenarios, i.e., training from scratch, LoRA finetuning with a rank of 8, and fine-tuning of all the model parameters. Fine-tuned models always outperforms models trained from scratch, possibly because of the reduced number of epochs required during fine-tuning to reach a small error with respect to a model trained from scratch. LoRA finetuning provides slightly smaller errors on forces with respect to full fine-tuning, while at the same time being faster, requiring the update of a reduced number of parameters.

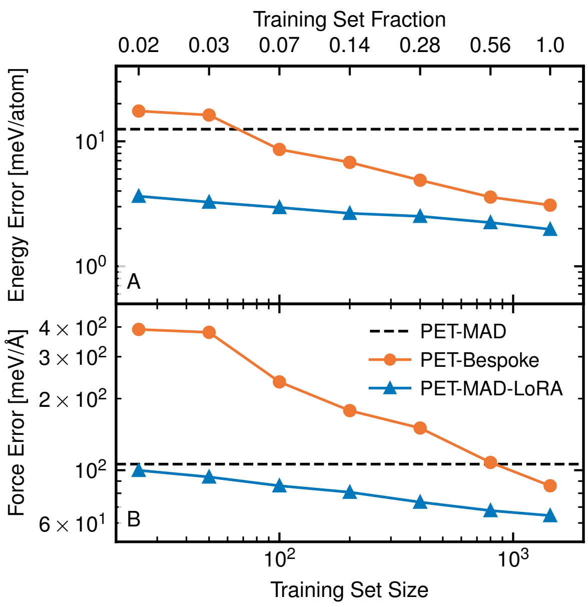

Supplementary Figure 13 Learning curves (forces) for the Succinic Acid dataset. The results compare training a bespoke model from scratch (blue line) and LoRA finetuning (orange line) with LoRA rank 8 against the base PET-MAD model predictions error (black dotted line).

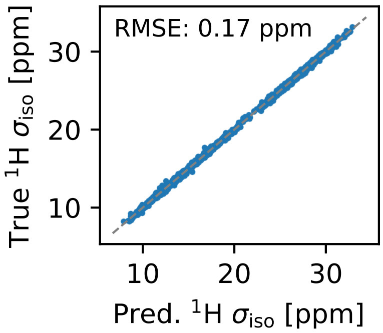

Supplementary Figure 14 Accuracy of the chemical shieldings prediction. Parity plot of the auxiliary model constructed to predict chemical shieldings in succinic acid crystals.

12 Non-conservative MD

We perform molecular-dynamics simulations of the molten phase of the ionic liquid 1-Butyl-3-methylimidazolium chloride (BMIM-Cl), starting from configurations equilibrated at 500 K. We compare simulations using (i) the standard, conservative forces computed as derivatives of the PET-MAD energy head; (ii) the non-conservative,

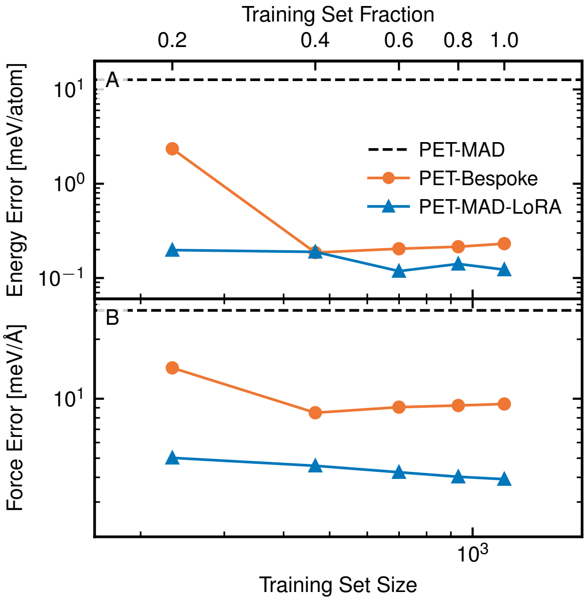

Supplementary Figure 15 Learning curves for the BTO dataset. The results compare training a bespoke model from scratch (blue line) and LoRA finetuning (orange line) with LoRA rank 8 against the base PET-MAD model predictions error (black dotted line).

direct force prediction head (which is approximately 2 times faster); (iii) a multiple-time-stepping setup in which the conservative forces are evaluated every 8 steps to correct the non-conservative trajectory (which is approximately 1.8 times faster than conservative MD). We refer the reader to Ref. [18] for a general discussion of the advantages and problems of direct force prediction.

We first perform constant-energy simulations, using a time step of 0.5 fs and a velocity Verlet integrator. When using a conservative force calculation, the kinetic temperature of the atoms as a function of time (Suppl. Fig. 16a) fluctuates around the initial equilibrium temperature, as expected. The non-conservative head leads to fast, catastrophic drift of the kinetic energy, that quickly leads to molecular dissociation and completely unphysical outcomes. A multiple-time-stepping strategy, instead, yields a stable trajectory, that is entirely consistent with conservative MD. As shown for other non-conservative potentials in Ref. [18], using an aggressive global thermostat avoids a drift in the overall temperature, but breaks energy conservation, leading to different chemical species (or more broadly, different degrees of freedom) reaching a different steady-state kinetic energy (Suppl. Fig. 16b). Even in this case, a multiple-time-stepping simulation avoids these artifacts, while retaining most of the computational advantage.

Multiple time step calculations also yield static and dynamic properties that are consistent with those of a conservative calculation, as shown in Suppl. Fig. 17. Both the Cl-Cl correlation function (reporting on the local structure in the liquid) and the Cl diffusion (reporting on the ionic conductivity) are within the statistical uncertainty. On

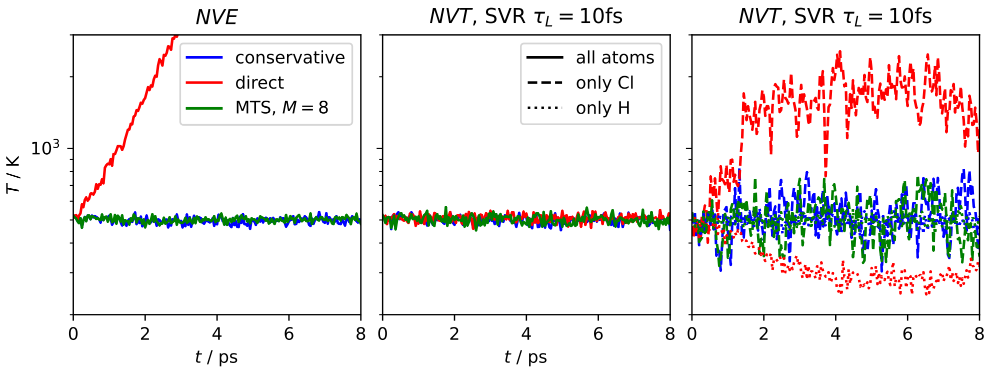

Supplementary Figure 16 Kinetic temperature profiles along MD simulations. The results are showing the temperature drift in the MD simulation using conservative force models (blue), direct force heads (red) and multiple time stepping (green). (left) Constant-energy simulations initialized from a structure equilibrated at 500 K; note the exponential drift occurring with non-conservative forces. (middle) Constant-temperature simulations using stochastic velocity rescaling, with a thermostat relaxation time τ_L =10 fs; direct-force simulations reach a steady-state with the overall temperature fluctuating around the target T = 500 K. (right) Temperature profiles for individual atomic types (Cl and H shown) demonstrate that the direct-force simulations are problematic even with aggressive global thermostatting; Cl reach a steady-state temperature above 2000 K, which is compensated by H (and other) atoms being well below the average temperature. Note that the larger kinetic energy fluctuations for Cl atoms, seen also for conservative trajectories, is normal and consistent with the fact that only a few Cl ions are present in the simulation box.

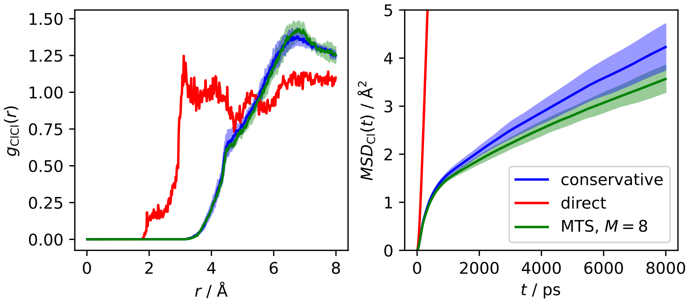

Supplementary Figure 17 Stability of the non-conservative MD simulations. Cl-Cl pair correlation function (left) and Cl mean square displacement (right) for simulations of BMIM-Cl at T = 500 K, using conservative force models (blue), direct force heads (red) and multiple time stepping (green). The shaded areas indicate a standard deviation computed from four independent trajectories.

the other hand, direct-force trajectories lead to extremely fast Cl diffusion (consistent with the much higher steady-state temperature) and the pair correlation function show the association of Cl dimers, another clear failure of the non-conservative sampling.

Supplementary References

[1] S Huber, M Bercx, N Hörmann, M Uhrin, G Pizzi, and N Marzari. Materials cloud three-dimensional crystals database (mc3d). *Materials Cloud Archive 2022.38*, 2022.

[2] Davide Campi, Nicolas Mounet, Marco Gibertini, Giovanni Pizzi, and Nicola Marzari. Expansion of the materials cloud 2d database. *ACS nano*, 17(12): 11268–11278, 2023.

[3] Manuel Cordova, Edgar A Engel, Artur Stefaniuk, Federico Paruzzo, Albert Hofstetter, Michele Ceriotti, and Lyndon Emsley. A machine learning model of chemical shifts for chemically and structurally diverse molecular solids. *The Journal of Physical Chemistry C*, 126(39):16710–16720, 2022.

[4] Colin R. Groom, Ian J. Bruno, Matthew P. Lightfoot, and Suzanna C. Ward. The Cambridge Structural Database. *Acta Crystallographica Section B Structural Science, Crystal Engineering and Materials*, 72(2):171–179, April 2016. ISSN 2052-5206. doi: 10.1107/S2052520616003954. URL https://journals.iucr.org/paper?S2052520616003954.

[5] Rose K Cersonsky, Maria Pakhnova, Edgar A Engel, and Michele Ceriotti. A data-driven interpretation of the stability of organic molecular crystals. *Chemical Science*, 14(5):1272–1285, 2023.

[6] Lowik Chanussot, Abhishek Das, Siddharth Goyal, Thibaut Lavril, Muhammed Shuaibi, Morgane Riviere, Kevin Tran, Javier Heras-Domingo, Caleb Ho, Weihua Hu, et al. Open catalyst 2020 (oc20) dataset and community challenges. *Acs Catalysis*, 11(10):6059–6072, 2021.

[7] Stefan Chmiela, Valentin Vassilev-Galindo, Oliver T Unke, Adil Kabylda, Huziel E Sauceda, Alexandre Tkatchenko, and Klaus-Robert Müller. Accurate global machine learning force fields for molecules with hundreds of atoms. *Science Advances*, 9(2):eadf0873, 2023.

[8] Hai-Chen Wang, Silvana Botti, and Miguel AL Marques. Predicting stable crystalline compounds using chemical similarity. *npj Computational Materials*, 7(1): 12, 2021.

[9] Janosh Riebesell, Rhys EA Goodall, Philipp Benner, Yuan Chiang, Bowen Deng, Alpha A Lee, Anubhav Jain, and Kristin A Persson. Matbench discovery–a framework to evaluate machine learning crystal stability predictions. *arXiv preprint arXiv:2308.14920*, 2023.

[10] Gianluca Prandini, Antimo Marrazzo, Ivano E. Castelli, Nicolas Mounet, and Nicola Marzari. Precision and efficiency in solid-state pseudopotential calculations. *npj Comput Mater*, 4(1):72, December 2018. ISSN 2057-3960. doi:

10.1038/s41524-018-0127-2.

[11] Filippo Bigi, Sanggyu Chong, Michele Ceriotti, and Federico Grasselli. A prediction rigidity formalism for low-cost uncertainties in trained neural networks. *Mach. Learn.: Sci. Technol.*, 5(4):045018, December 2024. ISSN 2632-2153. doi: 10.1088/2632-2153/ad805f.

[12] Matthias Kellner and Michele Ceriotti. Uncertainty quantification by direct propagation of shallow ensembles. *Mach. Learn.: Sci. Technol.*, 5(3):035006, September 2024. ISSN 2632-2153. doi: 10.1088/2632-2153/ad594a.

[13] Atsushi Togo, Laurent Chaput, and Isao Tanaka. Distributions of phonon lifetimes in brillouin zones. *Phys. Rev. B*, 91:094306, Mar 2015. doi: 10.1103/PhysRevB.91.094306. URL https://link.aps.org/doi/10.1103/PhysRevB.91.094306.

[14] Balázs Póta, Paramvir Ahlawat, Gábor Csányi, and Michele Simoncelli. Thermal conductivity predictions with foundation atomistic models, 2024. URL https://arxiv.org/abs/2408.00755.

[15] Antoine Loew, Dewen Sun, Hai-Chen Wang, Silvana Botti, and Miguel A. L. Marques. Universal machine learning interatomic potentials are ready for phonons, 2024. URL https://arxiv.org/abs/2412.16551.

[16] Lorenzo Gigli, Davide Tisi, Federico Grasselli, and Michele Ceriotti. Mechanism of charge transport in lithium thiophosphate. *Chemistry of Materials*, 36(3): 1482–1496, 2024. doi: 10.1021/acs.chemmater.3c02726. URL https://doi.org/10.1021/acs.chemmater.3c02726.

[17] Arslan Mazitov, Maximilian A Springer, Nataliya Lopanitsyna, Guillaume Fraux, Sandip De, and Michele Ceriotti. Surface segregation in high-entropy alloys from alchemical machine learning. *J. Phys. Mater.*, 7(2):025007, April 2024. ISSN 2515-7639. doi: 10.1088/2515-7639/ad2983.

[18] Filippo Bigi, Marcel Langer, and Michele Ceriotti. The dark side of the forces: assessing non-conservative force models for atomistic machine learning. *arXiv preprint arXiv:2412.11569*, 2024.
